# Alteration of Cell Membrane Permeability by Cetyltrimethylammonium Chloride Induces Cell Death in Clinically Important *Candida* Species

**DOI:** 10.3390/ijerph20010027

**Published:** 2022-12-20

**Authors:** Ravi Jothi, Ravichellam Sangavi, Veerapandian Raja, Ponnuchamy Kumar, Shunmugiah Karutha Pandian, Shanmugaraj Gowrishankar

**Affiliations:** 1 Department of Biotechnology, Science Campus, Alagappa University, Karaikudi 630 003, Tamil Nadu, India; 2 Center of Emphasis in Infectious Diseases, Department of Molecular and Translational Medicine, Paul L. Foster School of Medicine, Texas Tech University Health Sciences Center, El Paso, TX 79905, USA; 3 Department of Animal Health and Management, Science Campus, Alagappa University, Karaikudi 630 003, Tamil Nadu, India

**Keywords:** *Candida albicans*, clinical biofilms, fungal infections, CTAC, disinfectant

## Abstract

The increased incidence of healthcare-related *Candida* infection has necessitated the use of effective disinfectants/antiseptics in healthcare settings as a preventive measure to decontaminate the hospital environment and stop the persistent colonization of the offending pathogens. Quanternary ammonium surfactants (QASs), with their promising antimicrobial efficacy, are considered as intriguing and appealing candidates for disinfectants. From this perspective, the present study investigated the antifungal efficacy and action mechanism of the QAS cetyltrimethylammonium chloride (CTAC) against three clinically important *Candida* species: *C. albicans*, *C. tropicalis*, and *C. glabrata*. CTAC exhibited phenomenal antifungal activity against all tested *Candida* spp., with minimum inhibitory concentrations (MIC) and minimum fungicidal concentrations (MFC) between 2 and 8 µg/mL. The time–kill kinetics of CTAC (at 2XMIC) demonstrated that an exposure time of 2 h was required to kill 99.9% of the inoculums in all tested strains. An important observation was that CTAC treatment did not influence intracellular reactive oxygen species (ROS), signifying that its phenomenal anticandidal efficacy was not mediated via oxidative stress. In addition, sorbitol supplementation increased CTAC’s MIC values against all tested *Candida* strains by three times (8–32 μg/mL), indicating that CTAC’s possible antifungal activity involves fungus cell membrane destruction. Interestingly, the increased fluorescence intensity of CTAC-treated cells in both propidium iodide (PI) and DAPI staining assays indicated the impairment of cell plasma membrane and nuclear membrane integrity by CTAC, respectively. Additionally, CTAC at MIC and 2XMIC was sufficient (>80%) to disrupt the mature biofilms of all tested spp., and it inhibited the yeast-to-hyphae transition at sub-MIC in *C. albicans*. Finally, the non-hemolytic activity of CTAC (upto 32 µg/mL) in human blood cells and HBECs signified its non-toxic nature at the investigated concentrations. Furthermore, thymol and citral, two phytocompounds, together with CTAC, showed synergistic fungicidal effectiveness against *C. albicans* planktonic cells. Altogether, the data of the present study appreciably broaden our understanding of the antifungal action mechanism of CTAC and support its future translation as a potential disinfectant against *Candida*-associated healthcare infections.

## 1. Introduction

Healthcare-associated *Candida* infections have increased globally in recent years, presenting a serious concern due to the harm they cause patients, particularly those who stay in hospital for extended periods of time with compromised immune systems [1]. Notably, the estimated mortality rate of nosocomial candidemia accounts for nearly 49% of all deaths, which is unusually high for an infectious disease and even higher than the patients’ base disease death rate [2]. Negligence or the poor management of *Candida* outbreaks in hospital environments have resulted in the persistent colonization of the pathogen and disease transmission. On the other hand, a precise diagnosis of *Candida* infection and the administration of antifungal medications are not always possible in the group of patients with high mortality [3]. Therefore, ensuring adequate disinfectant/antiseptic procedures as a prophylactic step to decontaminate the hospital environment, including medical devices, is highly recommended, as it will in turn eventually prevent the persistent colonization of *Candida* species [4].

Quaternary ammonium surfactants (QASs) are the most ubiquitous chemical compounds, possessing a hydrophilic quaternary ammonium head group and one or two hydrophobic tail groups [5]. This amphiphilic nature lends QASs a variety of desirable qualities, allowing them to be used in various medicinal applications *viz*., antiseptics, surfactants, and anesthetics [6]. The broad-spectrum antimicrobial action of surfactants has been exploited since the mid-1930s, and they are now used as disinfectants/antiseptics in numerous general hygiene products [7]. Despite their wide range of utility, there remains much to be understood about their mechanistic action, which is imperative for the logical design of optimum formulations to hinder pathogens’ resistance-development tactics. The alarming global issue of the emergence of multidrug resistance further necessitates a complete understanding of the antimicrobial action mechanisms of drugs prior to their recommendation as disinfectants in healthcare settings [8].

CTAC is along-chain QAS that has been chiefly used in the manufacturing of antistatic fixatives for permanent waving, hair lotions, shampoos, and styling creams [9]. Most importantly, CTAC has commonly been used under its trade name ‘Genamin’ in the manufacturing of various cosmetic products, which signifies its ecological and toxicological safety [10]. Given its prominence, CTAC has been deployed in myriad pharmaceutical applications, i.e., as a micelle in drug delivery systems (especially in tumor therapy) and as a stabilizing agent for synthesizing nanoparticles [11,12].

An earlier report by Oliva et al. (2014) demonstrated well the antibacterial and antifungal efficacies of CTAC against *Lactobacillus fermentum* and *Saccharomyces cerevisiae*, respectively [13]. A study by Lyon et al. (2011) also documented the antifungal and antivirulence efficacies of CTAC against the growth and other virulence properties, *viz*., biofilm generation, yeast-to-hyphae transition, and proteinase production, of five *Candida* species [14]. Nevertheless, the mechanism underlying the antifungal action of CTAC against *Candida* species has not yet been investigated. It has traditionally been proposed that the antimicrobial activity of surfactants is achieved through altering the biological membrane structure of the target organisms [15]. It has also been suggested that the negative charge of cationic surfactants interferes with the positively charged microbial cell membrane to exert biocidal action [16]. However, this may not be the rule of thumb for all surfactants; hence, a thorough understanding of how CTAC affects the development of *Candida* species is essential before its deployment in any kind of formulation (including disinfectants).

Therefore, in the present study, we attempted to determine the antifungal mechanism of action of CTAC against three clinically significant *Candida* species, *C. albicans*, *C. tropicalis*, and *C. glabrata*, by assessing intracellular ROS and cell and nuclear membrane integrity and conducting sorbitol and ergosterol assays. Moreover, synergistic combinatorial therapy is considered a valid and pragmatic strategy for identifying drugs with novel modes of action. This method could potentially decrease single-drug dosages, leading to increased therapeutic efficacy and, subsequently, lowering the toxicity related to high doses [3,16]. Hence, the present study was also intended to assess the synergistic interactions of certain known phytochemicals with CTAC for enhanced antifungal efficacy.

## 2. Materials and Methods

### 2.1. Strains and Culture Conditions

The test organisms *C. albicans* (ATCC 10231), *C. glabrata* (MTCC 184), and *C. tropicalis* (MTCC3019) were purchased from Himedia India. The fungi were maintained in Sabouraud dextrose agar (SDA) plates and routinely cultured in yeast extract peptone dextrose (YEPD) broth at 37 °C until used in experiments. A 3 h culture with 0.1 optical density (OD) (1 × 10^6^ CFU/mL) was used as the inoculum to perform all in vitro assays.

### 2.2. Compound Preparation

The active drug CTAC was obtained from Sigma-Aldrich. The stock solution was prepared to a final concentration of 10 mg/mL using water as a solvent. Finally, the stock was stored at 4 °C for further use.

### 2.3. Determination of Minimal Inhibitory Concentrations (MICs)

The MIC of CTAC against *C. albicans*, *C. glabrata*, and *C. tropicalis* was determined using a broth microdilution assay as demonstrated by Gowrishankar et al. [17]. In brief, 1 mL of YEPD medium was dispensed into a 24-well Microtitre Plate (MTP). The CTAC solution (2048 μg/mL) was then added to the first well, which contained 2 mL of YEPD broth, and 1 mL of medium was serially diluted in the following two wells to achieve a CTAC concentration ranging from 0 to 1024 μg/mL. Finally, 1% of the 3 h cultures (1 × 10^6^ CFU/mL) corresponding to each fungal strain was added into each well, and the plate was stored at 37 °C for 48 h. After incubation, the growth density was measured at 600 nm using a spectrophotometer (Spectra Max 3, Molecular Devices, San Jose, CA, USA). The wells containing YEPD medium with and without a culture devoid of the test compound served as the control and blank, respectively. The MIC was defined as the minimal concentration of CTAC that exhibited visible growth inhibition in YEPD broth.

### 2.4. Determination of Minimal Fungicidal Concentrations (MFCs)

To determine the MFC, the spread plate method was performed as described by Hafidi et al. [18]. A total of 100 µL of the aliquots from the wells that showed visible growth inhibition in the broth microdilution assay were subcultured on the YEPD agar plates. After incubation at 37 °C for 48 h, CFU was calculated. MFC was defined as the lowest concentration of CTAC that showed either no growth or less than 10 fungal colonies to obtain approximately 99–99.5% killing activity.

### 2.5. Time–Kill Kinetics

The time–kill rate of CTAC in terms of the viability of the test fungal strains was determined using the method described by Öz et al. [19]. The initial inoculums of each fungal strain were adjusted to 1 × 10^6^ CFU/mL in YEPD broth before being treated with CTAC at MIC and 2XMIC. The YEPD medium containing fungal culture without CTAC served as the control. The suspensions were then incubated at 37 °C. At the predetermined time intervals (1 to 12 h with a 1 h time interval), 200 µL aliquots from each cell suspension were transferred into 96-well MTPs to measure the optical density, and 100 µL aliquots were spread over the YEPD agar plates to measure the CFU. In addition, 2 µL aliquots were spotted on YEPD agar plates. Subsequently, the agar plates were incubated at 37 °C for 48 h for CFU counting.

### 2.6. Sorbitol Assay

To determine the possible effect of CTAC on the cell walls of *Candida* species, a sorbitol protection assay was performed [20]. The MIC of CTAC in the presence and absence of sorbitol was determined using the abovementioned broth microdilution assay. Briefly, 1% of the test fungal strains was inoculated into 1 mL of YEPD medium supplemented with CTAC at various concentrations (0–1024 µg/mL) in addition to 0.8 M sorbitol. The plates were incubated at 37 °C, and the growth density was measured at 2 and 7 days.

### 2.7. Ergosterol Assay

In order to evaluate the possible mode of action of CTAC on fungal cell membrane sterol (ergosterol), an MIC assay was performed in the presence and absence of exogenous ergosterol using amphotericin B as a positive control [21]. Briefly, the test compound CTAC and amphotericin B were serially diluted from 0 to 1024 µg/mL and from 0 to 64 µg/mL, respectively, in 24-well MTPs containing 1 mL of YEPD medium. The exogenous ergosterol was added into each well at a concentration of 400 µg/mL along with 1% fungal inoculum. The plates were incubated at 37 °C for 24 h.

### 2.8. ROS Estimation

To detect the intracellular ROS accumulation by *Candida* species during CTAC treatment, the fluorescent dye 2′,7′-dichlorofluorescein diacetate (DCFH-DA) was used [22]. In brief, a fungal suspension (1 × 10^6^ CFU/mL) was treated with CTAC at ½ MIC and MIC for 4 h. Then, the cell suspension was incubated with DCFH-DA (2 µg/mL) for 30 min. The cell pellet obtained through centrifugation was dissolved in PBS and visualized under a florescence microscope to detect ROS accumulation.

### 2.9. DAPI Staining

The fungal cells that underwent nuclear fragmentation and condensation upon CTAC treatment were inspected using DAPI staining [23]. *Candida* strains treated with CTAC at ½ MIC and MIC for 4 h were stained by DAPI (1 μg/mL) for 30 min in the dark. After incubation, the cells were harvested through centrifugation, washed with PBS, and visualized under a fluorescence microscope.

### 2.10. Effect of CTAC on Preformed Biofilm

The inhibitory efficacy of CTAC on the preformed biofilm of *Candida* spp. was evaluated using the crystal violet staining method [24]. In brief, the overnight culture of *Candida* stains was inoculated into 1 mL of spider medium supplemented with 10% hyphal-inducing medium (FBS) in a 24-well MTP and incubated at 37 °C for 48 h to allow biofilm formation. After incubation, the spent medium was discarded and replaced with fresh spider medium along with CTAC at various concentrations. The plates were incubated at 37 °C for 24 h. Further, the non-adherent planktonic cells on the MTP were removed by washing with sterile PBS. Then, the sessile cells on the bottom of the MTP were stained using 0.4% crystal violet for 15 min (HiMedia, India). Any excess stain was removed by washing with sterile water. After 15 min of destaining with 15% glacial acetic acid, the amount of crystal violet bound to the biofilm cells was quantified spectrophotometrically at 570 nm. The percentage of biofilm inhibition was determined using the following formula.

The relative biofilm inhibition: % of biofilm inhibition = ((control OD570 nm − treated OD570 nm)/control OD570 nm) × 100.

The biofilm inhibitory concentration (BIC) was defined as the minimal concentration of CTAC that brought about 90% biofilm inhibition without affecting cellular viability.

### 2.11. Inhibitory Efficacy of CTAC on C. albicans Yeast-to-Hyphae Transition

To assess the impact of CTAC (at sub-MIC) on *C. albicans*’ transition from yeast to hyphal, a hyphal growth experiment in liquid media was conducted according to Bar-Yosef et al.’s [25] recommendations. In brief, an overnight culture of *C. albicans* was used to inoculate 1 mL of spider broth supplemented with sub-MIC levels of CTAC (0.5, 1, and 2 µg/mL) and incubated for 24 h at 37 °C. After incubation, the changes in the ratio of yeast to hyphae cells were further analyzed via a light microscope at 200× magnification (Nikon Eclipse 80i, Tokyo, Japan).

### 2.12. Erythrocyte Lysis Assay

Fivemicroliters of human blood was collected in a sterile 15 mL falcon tube containing sodium citrate as an anticoagulant agent [26]. The blood sample was centrifuged at 3000 rpm for 5 min. The residual erythrocyte was collected by discarding the culture supernatant and dissolved in PBS at the final concentration of 2%. Then, 100 µL erythrocyte suspension was incubated with different concentrations (MIC, 1XMIC, 2XMIC, and 4XMIC) of CTAC at 37 °C for 1 h. After incubation, hemolytic activity was measured by reading the supernatant at 415 nm.

### 2.13. Checkerboard Assay

The combined effect of CTAC on the antifungal activity of borneol, coumarin, eugenol, citral, and thymol was investigated as previously described by Muthamil et al. [27], with slight modifications. Five phytocompounds (borneol, coumarin, eugenol, citral, and thymol) were used in this experiment. CTAC and the five test phytocompounds were combined at five concentrations lower than their respective MIC values in 48-well microplates. Subsequently, a mid-log phase of *C. albicans* culture (1 × 10^7^ CFU/mL) was added to each well. After 24 h incubation at 37 °C, the MIC of each drug alone and in combination was determined by reading at 600 nm using a spectrophotometer. The combined effect was expressed as the fractional inhibitory concentration index (FICI). The FICI values were determined using the following formula.

FICI = (MIC of drug A in combination/MIC of drug A alone) + (MIC of drug B in combination/MIC of drug B alone). FICI values of ≤0.5, >0.5, and ≤4.0 were denoted as synergistic, non-interactive, and antagonistic, respectively.

### 2.14. Toxicity of CTAC on Human Buccal Epithelial Cells (HBECs)

The safety and toxicity of CTAC towards human buccal epithelial cells (HBECs) were investigated [28]. Briefly, the HBECs were extracted from healthy individuals by gently stroking a sterile swab on the mucosal surface of the cheeks. The sterile swabs were then suspended in sterile PBS, and a pellet was obtained by centrifugation before being washed three times with sterile PBS. Then, 5.0 × 10^5^ cell suspensions (counted using an automatic cell counter) were exposed to various concentrations of CTAC (2, 4, 8, 16, and 32 μg mL^−1^) for 20 min at 37 °C. Hydrogen peroxide was used as the positive control. Subsequent to incubation, the cells were stained with crystal violet and examined under a microscope (Nikon Eclipse Ts2R, Tokyo, Japan) to determine any morphological changes brought on by the CTAC.

### 2.15. Statistical Analysis

All the experiments were carried out in biological triplicate with at least two experimental replicates, and the data are presented as mean ± standard deviation. To evaluate statistical differences between controls and treated samples, one-way analysis of variance (ANOVA) and Dunnett’s post hoc test were performed using SPSS statistical software 17.0. Significance was represented by *p* ≤ 0.05 and <0.01, respectively.

## 3. Results and Discussion

The broad-spectrum antimicrobial properties of cationic QASs allow them to be used in many fields, such as agriculture, medicine, and industry. Due to their amphiphilic nature, these compounds have demonstrated a phenomenal ability to adsorb on many surfaces and to cover them with a layer [29]. This causes microorganisms to have a minimal possibility of adhering to surfaces coated with cationic surfactants [30]. QASs with the aforementioned characteristics are particularly beneficial in light of the documented recalcitrant *Candida* biofilms that resist several antifungal agents. In particular, these compounds are advantageous in the management of infectious diseases in healthcare settings.

Hospital-acquired candidemia has emerged as a significant infection due to not only its apparently increasing incidence but also its high fatality rate [31]. Since diagnosis and the administration of treatment options are not always feasible in patients at high risk, the prevention of disease transmission is viewed as an effective approach for controlling infectious diseases in hospital settings [32]. Due to the high prevalence of disease transmission through healthcare-related materials, decontaminating hospital environments with potent disinfectants and antiseptics is strongly advised [33].

QASs, with their promising antimicrobial efficacy, have been considered as intriguing and appealing candidates for disinfectants above other synthetic chemicals [5,6].From this perspective, the present study investigated the antifungal efficacy and action mechanism of the QAS CTAC against three clinically important *Candida* species, *viz*., *C. albicans*, *C. tropicalis*, and *C. glabrata*.

### 3.1. Low Conentrations of CTACExhibit Fungicidal Action against Candida Species

**Figure 1:**
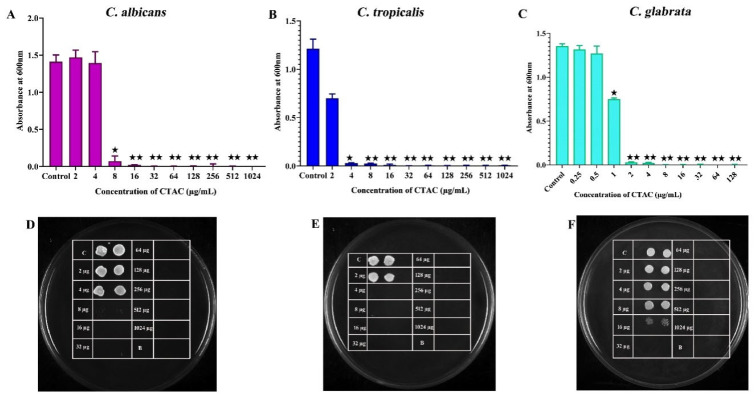
Determination of MIC of CTAC against *C. albicans*, *C. tropicalis*, and *C. glabrata*. (**A**–**C**) growth OD of fungal strains was measured through spectrophotometry after 24 h of treatment with CTAC at concentrations ranging from 2 to 1024 g/mL and 0.25 to 128 g/mL for *C. albicans*, *C. tropicalis*, and *C. glabrata*, respectively. (**D**–**F**) The growth inhibition of fungal strains was authenticated by the spot method. * and ** represent statistical significance at *p* < 0.05 and *p* < 0.01, respectively.

The antifungal activity of CTAC was initially evaluated by determining the MIC and MFC against three tested *Candida* species. It is widely accepted that developing a novel antifungal agent is more challenging because of the eukaryotic cell membranes produced by pathogenic fungi [34].CTAC’s MIC against *C. albicans*, *C. tropicalis*, and *C. glabrata* was determined to be 8, 4, and 2 μg/mL, respectively. The obtained growth-inhibition profiles upon treatment with CTAC are represented in Figure 1 as graphs and spot assays. The test results showed that *C. glabrata* (MIC 2 µg/mL) was more sensitive to CTAC than *C. albicans* and *C. tropicalis*. The results of the antifungal assay demonstrated that CTAC’s antifungal efficacy was species-dependent. Prior research on the anticandidal activity of CTAC found that the MIC for all three strains studied (*C. albicans*, *C. tropicalis*, and *C. glabrata*) was 0.78 μg/mL [14]. This variation could have been due to the difference in the tested strains.

**Figure 2:**
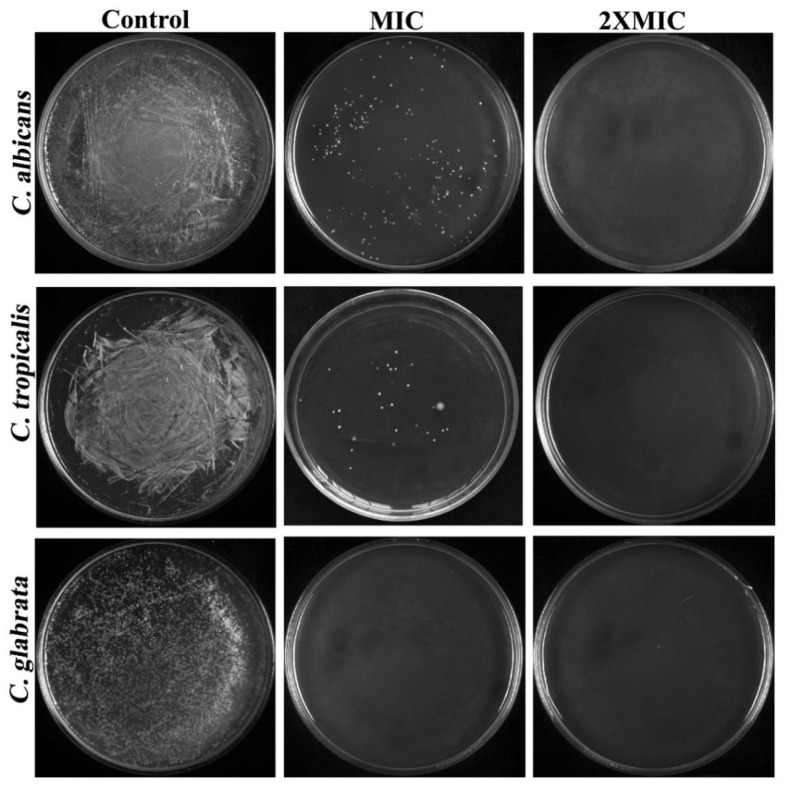
Determination of CTAC MFC against *C. albicans*, *C. tropicalis*, and *C. glabrata* through spread plate method.

To evaluate whether the test substance was fungicidal (MFC = MIC) or fungistatic (MFC > MIC), the ratio r = MFC/MIC was utilized [18] in accordance with the limits set by the most recent National Committee on Clinical Laboratory Standards (NCCLS). As depicted in Figure 2, the MFCs of CTAC for all tested organisms entirely coincided with their appropriate MICs, which signifies the CTAC’s fungicidal nature of killing *Candida* strains. It was inferred from the MIC and MFC of CTAC that the promising fungicidal efficacy of CTAC at very low concentrations might lend an edge for considering CTAC as an important disinfectant candidate in infection controlling process at hospitals.

**Figure 3:**
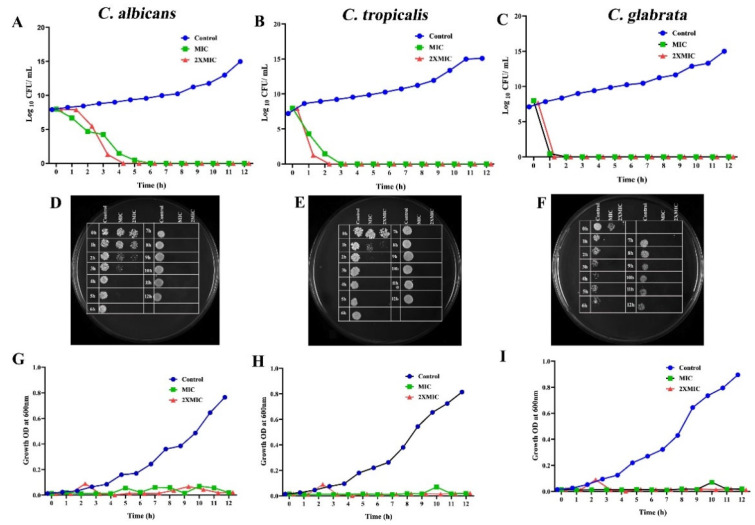
Time–kill kinetics of different concentrations of CTAC (MIC and 2XMIC) according to planktonic growth of tested *Candida* strains. (**A**–**C**) Graph plotted by comparing CFU/mL of fungal strains to duration of CTAC exposure. (**G**–**I**) Time–kill kinetics graph plotted by considering growth OD of fungal strains compared to duration of CTAC exposure. The fungal strain devoid of compound served as growth control. (**D**–**F**) Killing ability of CTAC over time was authenticated by the spot method.

Based on the MIC, the killing rate of CTAC at different time intervals was estimated by subjecting the fungal strains to time–kill kinetics experiments. In order to find out the dynamic relationship between the concentration of CTAC and its efficacy over time, the viable cell count, growth OD conferred by CFU, and spectrometric analysis were all taken into consideration. The CTAC exposure time (at MIC and 2XMIC) versus log_10_ CFU/mL and growth OD are shown in Figure 3. From the graph, it is obvious that CTAC’s MIC showed fungistatic action at up to 2 h of exposure, as the initial inoculum was reduced by less than 3 log_10_ CFU/mL. However, fungicidal action was observed at the MIC of CTAC (a decrease in initial inoculum CFU/mL > 3 log_10_) when the exposure time was over 3 h. It was evident from the time–kill kinetics that the concentration and length of exposure to the drug compound were the only factors that determined the transition between fungistatic and fungicidal activity. The findings of this experiment were consistent with those of Leite et al.’s [20] investigation, wherein the authors revealed that the transition between fungistatic and fungicidal effects depended on the concentration of citral. As represented in Figure 3, a complete reduction in *Candida* strain viability was observed after4 h of exposure for CTAC at MIC and 2 h at 2XMIC.

### 3.2. Elucidation of Fungicidal Action Mechanism of CTAC

#### Sorbitol Increased CTAC’s MIC against *Candida* Species, but Ergosterol Did Not

Despite the widespread use of cationic surfactants, the mechanism underlying their microbial cell disruption activity remains unclear. Understanding the mechanisms of action of agents, whether natural or synthetic, aids in the development of effective therapeutic approaches, particularly in the case of [35]. Generally, it is believed that surfactants affect biological membrane structures to exhibit toxicity towards pathogens [15,16]. Therefore, the action of CTAC on fungal cell walls was investigated by performing an MIC assay in the presence of sorbitol, an osmotic protectant used to stabilize fungal protoplasts; in such an assay, it is expected that the MIC of a drug that acts on cell walls will increase in the medium containing osmotic support [21]. The MIC values of CTAC in the presence of sorbitol against *Candida* strains are summarized in Table 1. The results revealed that adding sorbitol to the medium for two days raised the MIC of CTAC by up to three times compared to the medium without sorbitol (8–32 μg/mL), suggesting that the fungicidal action of CTAC could be carried out by targeting cell wall synthesis or assembly.

Further, it was hypothesized that CTAC targets candidal cell well biosynthesis upon interference with ergosterol (a basal component in the fungal cell membrane that plays a crucial role in permeability and fluidity). Hence, to prove this speculation, we performed an experiment involving the introduction of exogenous ergosterol into the medium containing CTAC and *Candida* species. Due to its unique characteristics, ergosterol serves as a target for most conventional antifungal drugs, for instance, polyenes and azoles [36]. The addition of exogenous ergosterol prevents the binding of compounds to the ergosterol of the cell membrane and, in turn, increases the MIC of compounds that show antifungal activity through binding to ergosterol [37]. Thus, the MIC of CTAC and amphotericin B (positive control) in the presence of exogenous ergosterol was evaluated. As seen in Table 1, there was no significant change observed in the MIC of CTAC in the presence of ergosterol, while the MIC value of amphotericin B increased up to 16-fold, suggesting that the mode of action of CTAC does not involve binding with ergosterol. This data reinforced the strong interaction of polyene compound -amphotericin B on fungal sterol as ascertained by Lima et al. [37].

### 3.3. CTAC (at MIC) Does Not Trigger the Production of ROS

**Figure 4:**
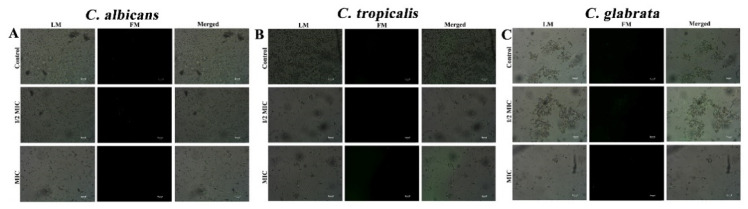
Assessment of intracellular ROS levels in candida cells such as (**A**) *C. albicans*, (**B**) *C. tropicalis* and (**C**) *C. glabrata* using DCFH-DA. LM—light microscopic images; FM—florescence micrograph; merged—merged fluorescence and light micrograph. Magnification—200×; scale bar—50 μm.

Previous studies on cationic multifunctional surfactants have implied that they enter cells and alter cell metabolism, notably by inducing the generation of superoxide anions and leading to oxidative stress [16]. ROS production occurs due to disturbances in a variety of cellular processes, including proliferation, inflammation, aging, and death [38]. Moderate levels of ROS are also generated in physiological responses as part of defensive mechanisms and signaling processes. Most often, the increased production of ROS that occurs during the antibacterial activity demonstrated by drug compounds leads to the induction of apoptosis in the target organism [22]. Therefore, the ROS accumulation in the *Candida* strains during treatment with CTAC was investigated using a ROS indicator (a DCFH-DA fluorescent probe). The results showed that there was no observable difference detected in the florescence intensity of the cells treated with CTAC. As seen in Figure 4, micrographs of both the untreated control and the treated samples displayed no significant fluorescence intensity, signifying that the fungicidal action of CTAC was not achieved through triggering ROS accumulation in the tested *Candida* strains. On the contrary, the findings of Paluch et al. [16] demonstrated that cationic dicephalic surfactants induce strong oxidative stress and cause an increase in cell membrane permeability without compromising their continuity in *C. albicans* cells. Furthermore, it was shown that the most widely used surfactant, CTAB, dramatically increased the generation of both superoxide and hydrogen peroxide, exerting toxicity on *Escherichia coli* and *C. albicans* cells [39,40]. These reports on chemical surfactants suggest that cationic surfactants can differ in their modes of action. Therefore, elucidating the mechanisms of action of individual drugs before their application as clinical formulations is highly recommended.

### 3.4. PI Staining Showed Loss of Cell Membrane Integrity Caused by CTAC

**Figure 5:**
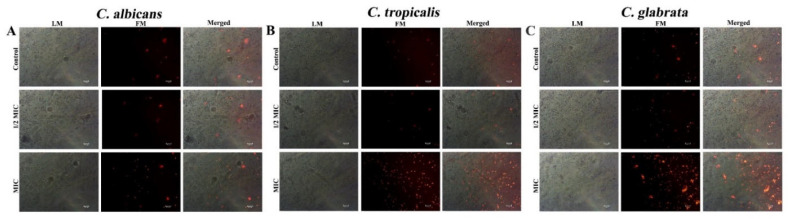
Cell membrane integrity of fungal strains such as (**A**) *C. albicans*, (**B**) *C. tropicalis* and (**C**) *C. glabrata* was assessed by PI staining after 30 min treatment with CTAC at 1/2MIC and MIC. LM—light microscopic images; FM—florescence micrograph; merged—merged fluorescence and light micrograph. Magnification—200×; scale bar—50 μm.

Although numerous studies have shown that cationic surfactants can interact with a variety of biomolecules, such as lipids and proteins, cell membrane disintegration is one of the ideal targets for mediating cell lysis [16,41]. As anticipated, the results of the sorbitol assay showed that CTAC affected the cell walls of all *Candida* strains. Cell membrane integrity was analyzed to gain more insights into the mechanism behind this effect. To this end, the membrane-impermeable DNA intercalating agent propidium iodide (PI) was employed. PI is considered an indicator of cell membrane integrity, as it specifically penetrates and stains DNA in dead cells or those with compromised cell membranes [42]. Cells with damaged membranes easily allow PI to enter and bind DNA, resulting in an increase in fluorescence intensity. As shown in Figure 5, after treatment with different concentrations of CTAC, an increase in the fluorescence intensity was observed compared to the untreated control cells, suggesting that CTAC significantly affected the cell membrane integrity in all tested *Candida* strains. We also observed that the PI-stained fungal cells displayed phosphatidylserine (PS) externalization, which is an early marker of apoptosis in fungi. Phosphatidylserine (PS) is normally found in the inner leaflet of the plasma membrane in healthy cells, whereas it is exposed on the outer leaflet in cells that have undergone apoptosis and necrosis [22]. Although CTAC exposure influenced cell membrane integrity, the lack of influence on ROS production in *Candida* cells upon CTAC treatment in turn signified that CTAC did not cause an apoptotic mode of *Candida* cell death.

### 3.5. CTAC (at MIC) CausesNuclear Membrane Damage

**Figure 6:**
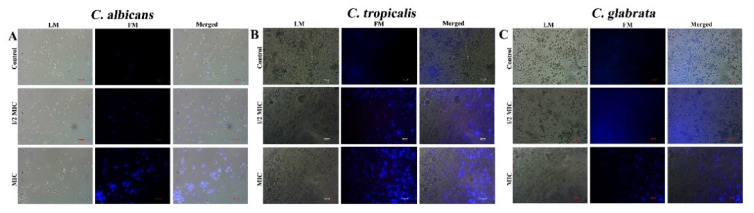
DNA damage induced by CTAC at different concentrations was analyzed by DAPI staining and examined under light and fluorescence microscopes. (**A**) *C. albicans*, (**B**) *C. tropicalis* and (**C**) *C. glabrata*. LM–light microscopic images; FM–florescence micrograph; merged—merged fluorescence and light micrograph. Magnification—200×; scale bar—50 μm.

Furthermore, DAPI staining was performed to determine whether CTAC could gain further access into cells, including the nucleus. DAPI is a fluorescent interacting stain that strongly binds to adenine–thymine-rich regions in DNA, where its fluorescence can be assessed. However, it is impermeable to the nuclear membrane and does not stain cells that have an intact nuclear membrane. If the nuclear membrane of a cell is damaged due to drug exposure, the permeability of DAPI is increased, resulting in a deep blue fluorescence that highlights nuclei with abnormal margins [23]. Fluorescence microscopic analysis revealed that *Candida* cells treated with CTAC exhibited more intense fluorescence in comparison with untreated cells, indicating nuclear condensation (Figure 6).

Even though PS externalization and nuclear condensation are recognized as hallmarks of apoptosis, the lack of influence on ROS production in CTAC-treated cells signified that the mode of cell death was ROS-independent. Overall, the experimental data suggested that the mechanism underlying the fungicidal action of CTAC was candidal cell membrane and nuclear membrane disruption, which eventually led to cell death in a ROS-independent fashion.

### 3.6. CTAC (at MIC) Diminishes Candida Virulence Attributes

Due to their inherent ability to form biofilms on different medical devices, *Candida* species have become import antifungal pathogens that cause nosocomial infections ranging from superficial to invasive in patients with compromised immune systems [43]. Although *Candida* species share a pathogenesis, the biofilm-forming ability varies greatly between species and depends on the host niche, surface, and other parameters [44]. The biofilm of *C. albicans* and *C. tropicalis* consists of yeast, pseudohyphae, and hyphae with extensive hyphal budding, whereas the biofilm of *C. glabrata* is made up of tightly packed multilayered yeast cells [45]. These variations illustrate the intricacy of the biofilm formation process and the challenge of developing a special method to eradicate every *Candida* biofilm.

**Figure 7:**
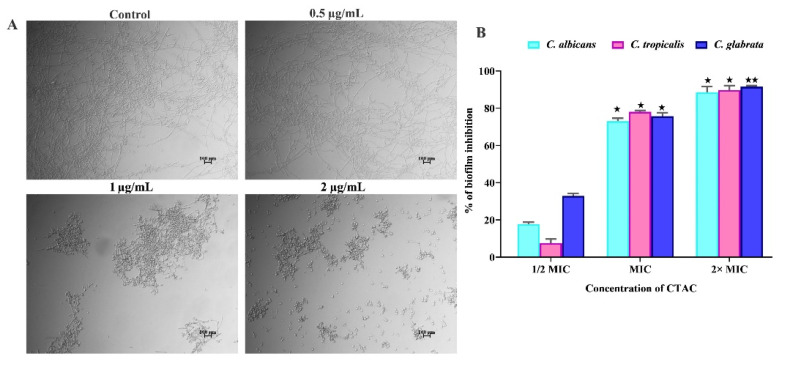
The influence of CTAC on virulence traits of *Candida* species. (**A**) Effect of CTAC (at sub-MIC) on yeast-to-hyphae transition in *C. albicans*. After 24 h incubation, samples were visualized under a light microscope (magnification—200×; scale bar—50 μm). (**B**) The evaluation of CTAC efficacy against mature biofilm production by *Candida* species through crystal violet staining. * and ** represent statistical significance at *p* < 0.05 and *p* < 0.01, respectively.

Moreover, when biofilms become established (referred to as mature biofilms), the implanted sessile cells become incredibly robust, rendering all antimicrobial treatments useless. In particular, the mature biofilms produced by *C. albicans* are very diverse in terms of the dispersion of their extracellular components. Mature biofilms represent a source of recurring infection, since they are very difficult to eliminate even with large dosages of antimicrobials [46]. Thus, the effect of CTAC on mature biofilms of *Candida* species (48 h) was assessed using the crystal violet staining method. The results revealed that the sub-MIC (1/2MIC) of CTAC was not sufficient to impair the preformed biofilms of *Candida* species. However, CTAC at MIC and 2XMIC effectively destroyed the biofilm, reducing it by over 75 and 90%, respectively (Figure 7). As the eradication of a mature biofilm thwarts its further development, CTAC could be particularly beneficial in light of the documented resistance of *Candida* biofilms to several antifungal agents in hospital settings.

In addition to biofilm formation, the yeast-to-hyphal transition of *C. albicans* and *C. tropicalis* is considered a putative virulence trait, as it closely regulates the other virulence traits for host cellular invasion [47]. Several investigators have suggested that molecules with high efficacy in inhibiting the yeast-to-hyphae transition represent attractive candidates for the treatment of candidiasis [25]. Hence, to examine the antihyphal efficacy of CTAC at sub-MIC levels (1–4 µg/mL), we performed a hyphal inhibitory assay using *C. albicans* alone. When cultured in liquid spider medium, the light micrographs of the untreated control groups exposed a dense network of twig-like hyphal formations (Figure 7). On the other hand, the micrographs of the CTAC-treated (at 4 μg/mL) groups displayed evenly scattered yeast cells. Moreover, subsequent sub-MIC doses (2 and 1 µg/mL) of CTAC did not alter the yeast-to-hyphae transition.

### 3.7. Non-Toxic Nature of CTAC Active Concentration forHuman Red Blood Cells and HBECs

**Figure 8:**
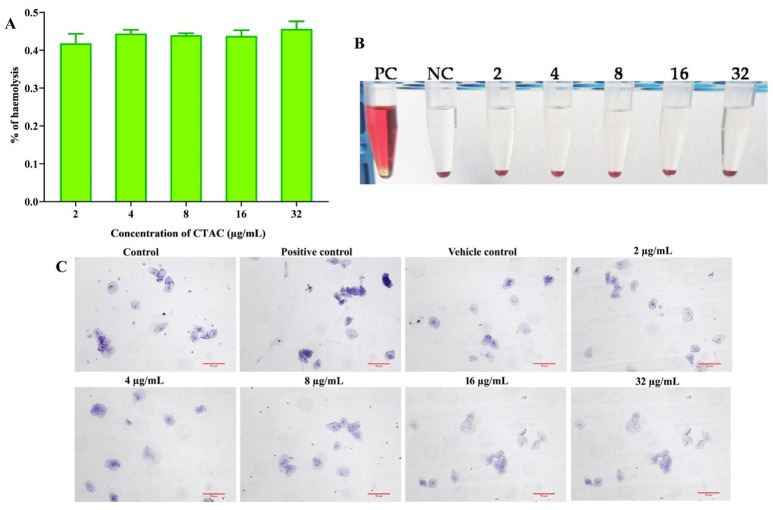
Assessment of CTAC cytotoxicity and HBECs. (**A**) Effect of CTAC on human red blood cells. No hemolytic activity was observed. The graph was plotted with absorbance using Triton X-100 as positive control, which seemed to completely lyse the total erythrocytes. (**B**) Representative image showing the non-hemolytic activity of CTAC. (**C**) No toxic effect was observed on HBECs when hydrogen peroxide and water were used as positive and vehicle control. Light micrograph magnification 200×, scale bar 50 μm.

An ideal bioactive molecule with clinical applicability must be non-toxic in nature. Therefore, CTAC (upto 32 μg/mL) was subjected to an in vitro cytotoxicity analysis involving human red blood cells and HBECs. At the tested concentrations, neither erythrocytes nor HBECs showed any harmful effects. The toxicity profiles of CTAC for erythrocytes and HBECs are shown in Figure 8. As seen in Figure 8A,B, exposure to CTAC at a concentration of upto 32 μg/mL did not lyse the erythrocytes, as the intact erythrocytes settled, leaving a clear PBS. On the other hand, the tube exposed to the positive control SDS (10%) exhibited a red color owing to the complete lysis of the red blood cells. In a similar fashion, CTAC did not cause any morphological changes in the HBECs, while the positive control hydrogen peroxide caused changes in the normal cells’ morphology (Figure 8C). Taken together, these findings demonstrated that CTAC could be considered safe for human application, as it did not cause any negative effects on human erythrocytes or HBECs. However, further studies to evaluate the toxicity profile of CTAC in in vivo models are warranted before its clinical application.

### 3.8. CTAC Exhibits Antifungal Synergism with Citral and Thymol

Combination therapy is a valid and pragmatic strategy for identifying drugs with novel modes of action. It can potentially decrease single-drug doses, leading to increased therapeutic efficacy and, subsequently, lowering the toxicity related to high doses. Additionally, the simultaneous targeting of two or more pharmacological targets may slow down the development of drug resistance [48]. For instance, the first ever synergistic combination used against invasive candidiasis was flucytosine and amphotericin B, in which the unanticipated side effect caused by flucytosine was compensated for by the addition of amphotericin B [49]. Moreover, recent reports have indicated that combinations of antifungal agents with newly identified phytochemicals exhibit high efficacy with low toxicity and a broad spectrum of action [50,51]. For example, a research group determined that the combined action of eugenol significantly reduced the SMIC (sessile MIC) of fluconazole by 32-fold [52]. In this milieu, the synergistic fungicidal activity of CTAC was investigated in combination with certain phytochemicals against *C. albicans*. The phytocompounds investigated, *viz*., thymol [53], citral [54], coumarin [22], eugenol [55], and borneol [56], were selected by considering the available literature pertaining to their activity against *Candida* species. First, the MICs of the investigated phytocompounds (thymol, citral, coumarin, eugenol, and borneol) were found to be 128, 128, 1024, 256, and 512 μg/mL, respectively.

**Figure 9:**
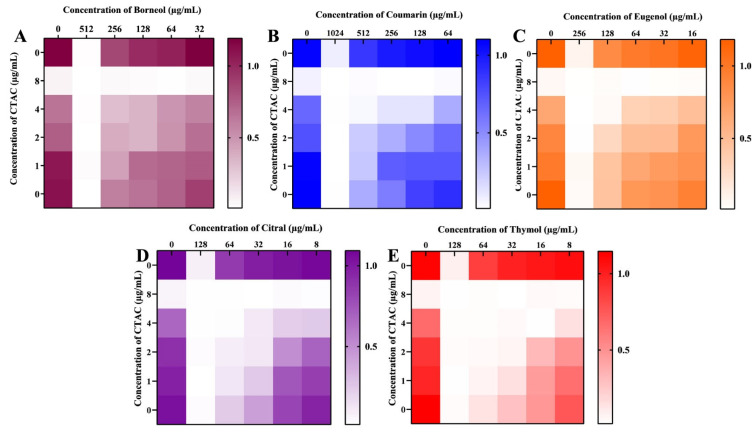
Synergistic antifungal effect of CTAC and the phytocompounds (**A**) borneol, (**B**) coumarin, (**C**) eugenol, (**D**) citral, and (**E**) thymol. Heatmap displays the growth OD measured in cells treated with phytocompounds alone or in combination with CTAC.

The synergistic effect of CTAC and these five phytocompounds on the growth of *C. albicans* was analyzed through a checkerboard assay. The synergism between the compounds was calculated by the FICI index. Among the five tested combinations, three showed no synergism: CTAC–borneol, CTAC–coumarin, and CTAC–eugenol (FICI indices >0.5). However, a significant synergistic effect was observed for the combination of citral and thymol with CTAC. The optimal synergistic concentrations of CTAC with citral and thymol were determined to be 2–32 and 1–32 μg/mL, with FICI values of 0.5 and 0.375, respectively. As depicted in Figure 9, the highest percentage inhibition caused by these two synergistic combinations, CTAC–citral (2–32 μg/mL) and CTAC–thymol (1–32 μg/mL), was in the range of 80–90. Conversely, the percentage of growth inhibition in cells treated with single doses of CTAC (1, 2 μg/mL), citral (32 μg/mL), and thymol (32 μg/mL) was found to be 23, 25, and 20, respectively. This result suggested that the synergistic antifungal efficacy of the identified combinations was higher than their efficacy when applied alone. Hence, the synergistic combination of CTAC with citral and thymol could be applied in disinfectant formulations, specifically to prevent *Candida*-associated infections in hospital settings.

**Figure 10:**
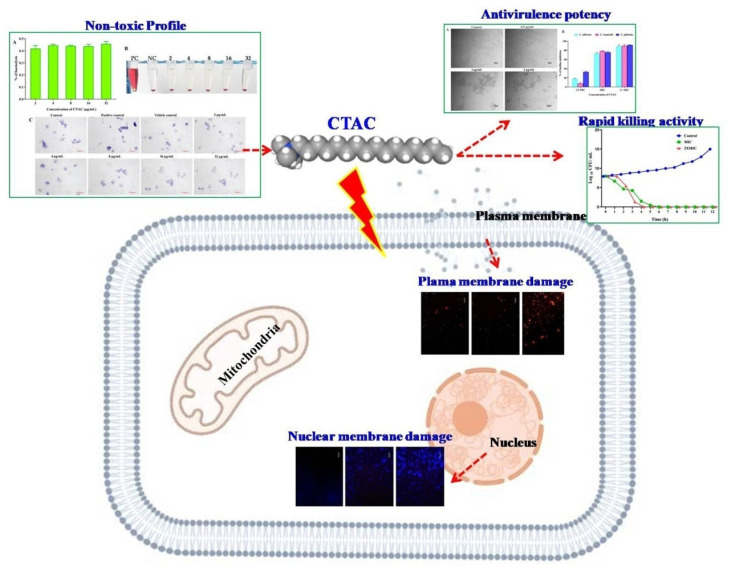
Overview of antifungal modes of action exhibited by CTAC against *Candida* species.

Altogether, the experiment of the present investigations proves the CTAC could be considered as effective disinfectant in healthcare settings. The overall mechanistic action of CTAC on *Candida* strains is illustrated in Figure 10.

## 4. Conclusions

Altogether, the current investigation demonstrated the potential antifungal efficacy of the cationic surfactant CTAC against the growth and other virulence traits of three clinically important *Candida* strains. Our results also shed more light on the underlying fungicidal action mechanisms of CTAC, which involve interference with cell and nuclear membranes. Moreover, the proven synergistic fungicidal and non-cytotoxic efficacy of CTAC supports its use in the development of new medical formulations, as it displays high pharmacological potential both alone and in combination with other phytocompounds for controlling *Candida* disease progression in clinical settings.

## Figures and Tables

**Table 1:** Minimal inhibitory concentration (MIC) values for CTAC against *Candida* species in the presence or absence of sorbitol or ergosterol.

Strain Name	MIC	MIC in the Presence of Sorbitol	MIC in the Presence of Ergosterol
*C. albicans*	8	32	8
*C. tropicalis*	4	16	4
*C. glabrata*	2	8	2

The MIC result are expressed as mode in μg/mL.

## Data Availability

Not applicable.

## References

[1] Bougnoux M.E., Brun S., Zahar J.R. (2018). Healthcare-associated fungal outbreaks: New and uncommon species, new molecular tools for investigation and prevention. Antimicrob. Resist. Infect. Control.

[2] Rodríguez-Leguizamón G., Fiori A., Lagrou K., Gaona M.A., Ibáñez M., Patarroyo M.A., Van Dijck P., Gómez-López A. (2015). New echinocandin susceptibility patterns for nosocomial *Candida albicans* in Bogotá, Colombia, in ten tertiary care centres: An observational study. BMC Infect. Dis..

[3] Karacaer Z., Oncul O., Turhan V., Gorenek L., Ozyurt M. (2014). A surveillance of nosocomial *candida* infections: Epidemiology and influences on mortalty in intensive care units. Pan Afr. Med. J..

[4] Rostami N., Alidadi H., Zarrinfar H., Ketabi D., Tabesh H. (2021). Interventional effect of nanosilver paint on fungal load of indoor air in a hospital ward. Can. J. Infect. Dis. Med. Microbiol..

[5] Zhang C., Cui F., Zeng G.M., Jiang M., Yang Z.Z., Yu Z.G., Shen L.Q. (2015). Quaternary ammonium compounds (QACs): A review on occurrence, fate and toxicity in the environment. Sci. Total Environ..

[6] Jiao Y., Niu L.N., Ma S., Li J., Tay F.R., Chen J.H. (2017). Quaternary ammonium-based biomedical materials: State-of-the-art, toxicological aspects and antimicrobial resistance. Prog. Polym. Sci..

[7] Jennings M.C., Minbiole K.P., Wuest W.M. (2015). Quaternary ammonium compounds: An antimicrobial mainstay and platform for innovation to address bacterial resistance. ACS Infect. Dis..

[8] Zarrinfar H., Kord Z., Fata A. (2021). High incidence of azole resistance among *Candida albicans* and *C. glabrata* isolates in Northeastern Iran. Curr. Med. Mycol..

[9] Colafemmina G., Palazzo G., Mateos H., Amin S., Fameau A.L., Olsson U., Gentile L. (2020). The cooling process effect on the bilayer phase state of the CTAC/cetearyl alcohol/water surfactant gel. Colloids Surf. A Physicochem. Eng. Asp..

[10] Mottier A., Pini J., Costil K. (2014). Effects of a POEA surfactant system (Genamin T-200^®^) on two life stages of the Pacific oyster, *Crassostrea gigas*. J. Toxicol. Sci..

[11] Slesiona N., Thamm S., Stolle HL K., Weißenborn V., Müller P., Csáki A., Fritzsche W. (2020). DNA-Biofunctionalization of CTAC-capped gold nanocubes. Nanomaterials.

[12] Tang M., Zhang P., Liu J., Long Y., Cheng Y., Zheng H. (2020). Cetyltrimethylammonium chloride-loaded mesoporous silica nanoparticles as a mitochondrion-targeting agent for tumor therapy. RSC Adv..

[13] Oliva Neto P.D., Lima FA D., Silva KC D., Silva DF D., Carvalho AF A., Santos C.D. (2014). Chemical inhibition of the contaminant *Lactobacillus fermentum* from distilleries producing fuel bioethanol. Braz. Arch. Biol. Technol..

[14] Lyon J.P., Dos Santos F.V., de Moraes PC G., Moreira L.M. (2011). Inhibition of virulence factors of *Candida* spp. by different surfactants. Mycopathologia.

[15] Hamouda T., Baker J.R. (2000). Antimicrobial mechanism of action of surfactant lipid preparations in enteric Gram-negative bacilli. J. Appl. Microbiol..

[16] Paluch E., Szperlik J., Lamch Ł., Wilk K.A., Obłąk E. (2021). Biofilm eradication and antifungal mechanism of action against *Candida albicans* of cationic dicephalic surfactants with a labile linker. Sci. Rep..

[17] Gowrishankar S., Pandian S.K. (2017). Modulation of *Staphylococcus epidermidis* (RP62A) extracellular polymeric layer by marine cyclic dipeptide-cyclo (l-leucyl-l-prolyl) thwarts biofilm formation. Biochim. Biophys. Acta Biomembr..

[18] Hafidi Z., El Achouri M., OSousa F.F., Perez L. (2022). Antifungal activity of amino-alcohols based cationic surfactants and in silico, homology modeling, docking and molecular dynamics studies against lanosterol 14-α-demethylase enzyme. J. Biomol. Struct. Dyn..

[19] Öz Y., Özdemir H.G., Gökbolat E., Kiraz N., Ilkit M., Seyedmousavi S. (2016). Time-kill kinetics and in vitro antifungal susceptibility of non-fumigatus *Aspergillus* species isolated from patients with ocular mycoses. Mycopathologia.

[20] Leite M.C.A., Bezerra A.P.D.B., Sousa J.P.D., Guerra F.Q.S., Lima E.D.O. (2014). Evaluation of antifungal activity and mechanism of action of citral against *Candida albicans*. Evid. Based Complement. Altern. Med..

[21] Leite M.C.A., de Brito Bezerra A.P., de Sousa J.P., de Oliveira Lima E. (2015). Investigating the antifungal activity and mechanism (s) of geraniol against *Candida albicans* strains. Med. Mycol..

[22] Jia C., Zhang J., Yu L., Wang C., Yang Y., Rong X., Xu K., Chu M. (2019). Antifungal activity of coumarin against *Candida albicans* is related to apoptosis. Front. Cell. Infect. Microbiol..

[23] Hao B., Cheng S., Clancy C.J., Nguyen M.H. (2013). Caspofungin kills *Candida albicans* by causing both cellular apoptosis and necrosis. Antimicrob. Agents Chemother..

[24] Jothi R., Sangavi R., Kumar P., Pandian S.K., Gowrishankar S. (2021). Catechol thwarts virulent dimorphism in *Candida albicans* and potentiates the antifungal efficacy of azoles and polyenes. Sci. Rep..

[25] Bar-Yosef H., Vivanco Gonzalez N., Ben-Aroya S., Kron S.J., Kornitzer D. (2017). Chemical inhibitors *of Candida albicans* hyphal morphogenesis target endocytosis. Sci. Rep..

[26] Turecka K., Chylewska A., Kawiak A., Waleron K.F. (2018). Antifungal activity and mechanism of action of the Co (III) coordination complexes with diamine chelate ligands against reference and clinical strains of *Candida* spp.. Front. Microbiol..

[27] Muthamil S., Balasubramaniam B., Balamurugan K., Pandian S.K. (2018). Synergistic effect of quinic acid derived from *Syzygiumcumini* and undecanoic acid against *Candida* spp. biofilm and virulence. Front. Microbiol..

[28] Priya A., Nivetha S., Pandian S.K. (2022). Synergistic Interaction of Piperine and Thymol on Attenuation of the Biofilm Formation, Hyphal Morphogenesis and Phenotypic Switching in *Candida albicans*. Front. Cell. Infect. Microbiol..

[29] Falk N.A. (2019). Surfactants as antimicrobials: A brief overview of microbial interfacial chemistry and surfactant antimicrobial activity. J. Surfactants Deterg..

[30] Paraszkiewicz K., Moryl M., Płaza G., Bhagat D., KSatpute S., Bernat P. (2021). Surfactants of microbial origin as antibiofilm agents. Int. J. Environ. Health Res..

[31] Niyas V.K., Rahulan S.D., Arjun R., Sasidharan A. (2021). ICU-acquired Candidemia in COVID-19 Patients: An Experience from a Tertiary Care Hospital in Kerala, South India. Indian Journal of Critical Care Medicine: Peer-reviewed. Off. Publ. Indian Soc. Crit. Care Med..

[32] Leas B.F., Sullivan N., Han J.H., Pegues D.A., Kaczmarek J.L., Umscheid C.A. (2015). Environmental Cleaning for the Prevention of Healthcare-Associated Infections.

[33] Haque M., McKimm J., Sartelli M., Dhingra S., Labricciosa F.M., Islam S., Jahan D., Nusrat T., Chowdhury T.S., Coccolini F. (2020). Strategies to prevent healthcare-associated infections: A narrative overview. Risk Manag. Healthc. Policy.

[34] Roemer T., Krysan D.J. (2014). Antifungal drug development: Challenges, unmet clinical needs, and new approaches. Cold Spring Harb. Perspect. Med..

[35] Davies J., Davies D. (2010). Origins and evolution of antibiotic resistance. Microbiol. Mol. Biol. Rev..

[36] Paquet M.J., Fournier I., Barwicz J., Tancrède P., Auger M. (2002). The effects of amphotericin B on pure and ergosterol-or cholesterol-containing dipalmitoylphosphatidylcholine bilayers as viewed by 2H NMR. Chem. Phys. Lipids.

[37] Lima I.O., de Medeiros Nóbrega F., de Oliveira W.A., de Oliveira Lima E., Albuquerque Menezes E., Afrânio Cunha F., de Fátima Formiga Melo Diniz M. (2012). Anti-*Candida albicans* effectiveness of citral and investigation of mode of action. Pharm. Biol..

[38] Pandey R., Gupta S., Shukla V., Tandon S., Shukla V. (2013). Antiaging, antistress and ROS scavenging activity of crude extract of *Ocimum sanctum* (L.) in *Caenorhabditis elegans* (Maupas, 1900). Indian J. Exp. Biol..

[39] Nakata K., Tsuchido T., Matsumura Y. (2011). Antimicrobial cationic surfactant, cetyltrimethylammonium bromide, induces superoxide stress in *Escherichia coli* cells. J. Appl. Microbiol..

[40] Yu Q., Zhang B., Ma F., Jia C., Xiao C., Zhang B., Li M. (2015). Novel mechanisms of surfactants against *Candida albicans* growth and morphogenesis. Chem. Biol. Interact..

[41] Vieira D.B., Carmona-Ribeiro A.M. (2006). Cationic lipids and surfactants as antifungal agents: Mode of action. J. Antimicrob. Chemother..

[42] Rosenberg M., Azevedo N.F., Ivask A. (2019). Propidium iodide staining underestimates viability of adherent bacterial cells. Sci. Rep..

[43] Gulati M., Nobile C.J. (2016). *Candida albicans* biofilms: Development, regulation, and molecular mechanisms. Microbes Infect..

[44] Nourizadeh N., Adabizadeh A., Zarrinfar H., Majidi M., Jafarian A.H., Najafzadeh M.J. (2019). Fungal biofilms in sinonasal polyposis: The role of fungal agents is notable?. J. Oral Maxillofac. Surg. Med. Pathol..

[45] Cavalheiro M., Teixeira M.C. (2018). *Candida* biofilms: Threats, challenges, and promising strategies. Front. Med..

[46] Chandra J., Kuhn D.M., Mukherjee P.K., Hoyer L.L., McCormick T., Ghannoum M.A. (2001). Biofilm formation by the fungal pathogen *Candida albicans*: Development, architecture, and drug resistance. J. Bacteriol..

[47] Mayer F.L., Wilson D., Hube B. (2013). *Candida albicans* pathogenicity mechanisms. Virulence.

[48] Cui J., Ren B., Tong Y., Dai H., Zhang L. (2015). Synergistic combinations of antifungals and anti-virulence agents to fight against *Candida albicans*. Virulence.

[49] Hatipoglu N., Hatipoglu H. (2013). Combination antifungal therapy for invasive fungal infections in children and adults. Expert Rev. Anti. Infect. Ther..

[50] Bisso B.N., Kayoka-Kabongo P.N., Tchuenguem R.T., Dzoyem J.P. (2022). Phytochemical Analysis and Antifungal Potentiating Activity of Extracts from Loquat (Eriobotrya japonica) against *Cryptococcus neoformans* Clinical Isolates. Adv. Pharmacol. Pharm. Sci..

[51] Ayaz M., Ullah F., Sadiq A., Ullah F., Ovais M., Ahmed J., Devkota H.P. (2019). Synergistic interactions of phytochemicals with antimicrobial agents: Potential strategy to counteract drug resistance. Chem. Biol. Interact..

[52] Khan Z., Ahmad S., Joseph L., Chandy R. (2012). *Candida dubliniensis*: An appraisal of its clinical significance as a bloodstream pathogen. PLoS ONE.

[53] De Castro R.D., de Souza TM P.A., Bezerra LM D., Ferreira GL S., de Brito Costa EM M., Cavalcanti A.L. (2015). Antifungal activity and mode of action of thymol and its synergism with nystatin against *Candida* species involved with infections in the oral cavity: An in vitro study. BMC Complement. Altern. Med..

[54] Silva CD B.D., Guterres S.S., Weisheimer V., Schapoval E.E. (2008). Antifungal activity of the lemongrass oil and citral against *Candida spp*. Braz. J. Infect. Dis..

[55] He M., Du M., Fan M., Bian Z. (2007). In vitro activity of eugenol against *Candida albicans* biofilms. Mycopathologia.

[56] Wang Y., Liu H., Zhan F. (2022). Effects of Natural Borneol on Germ Tube Formation and Preformed Biofilm Activity in *Candida albicans*. Nat. Prod. Commun..

